# 1-(6,8-Dibromo-2-methyl­quinolin-3-yl)ethanone

**DOI:** 10.1107/S1600536811037044

**Published:** 2011-09-17

**Authors:** R. Prasath, P. Bhavana, Seik Weng Ng, Edward R. T. Tiekink

**Affiliations:** a Department of Chemistry, BITS, Pilani – K. K. Birla Goa Campus, Goa 403 726, India; b Department of Chemistry, University of Malaya, 50603 Kuala Lumpur, Malaysia; c Chemistry Department, Faculty of Science, King Abdulaziz University, PO Box 80203 Jeddah, Saudi Arabia

## Abstract

Two independent mol­ecules,1 and 2, with similar conformations comprise the asymmetric unit in the title compound, C_12_H_9_Br_2_NO. The major difference between the mol­ecules relates to the relative orientation of the ketone–methyl groups [the C—C—C—C torsion angles are −1.7 (6) and −16.8 (6)° for mol­ecules 1 and 2, respectively]; in each case, the ketone O atom is directed towards the ring-bound methyl group. The crystal packing comprises layers of mol­ecules, sustained by C—H⋯O and π–π {ring centroid(C_6_) of molecule 2 with NC_5_ of molecule 1 [3.584 (3) Å] and NC_5_ of molecule 2 [3.615 (3) Å]} interactions. C—H⋯Br contacts also occur.

## Related literature

For background details and the biological applications of quinolines, see: Kalluraya & Sreenivasa (1998 ▶); Xiang *et al.* (2006 ▶). For a related structure, see: Prasath *et al.* (2011 ▶). For additional structure analysis, see: Spek (2009 ▶).
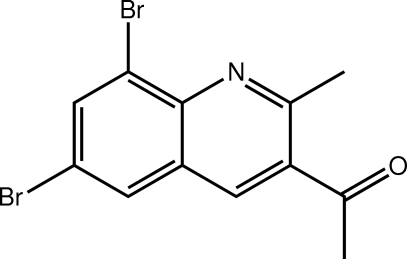


## Experimental

### 

#### Crystal data

C_12_H_9_Br_2_NO
*M* *_r_* = 343.02
Triclinic, 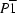

*a* = 9.7549 (5) Å
*b* = 11.1719 (6) Å
*c* = 11.5629 (5) Å
α = 99.043 (4)°
β = 93.330 (4)°
γ = 111.733 (5)°
*V* = 1146.69 (10) Å^3^
*Z* = 4
Cu *K*α radiation
μ = 8.78 mm^−1^
*T* = 100 K
0.25 × 0.20 × 0.15 mm

#### Data collection

Agilent SuperNova Dual diffractometer with an Atlas detector
Absorption correction: multi-scan (*CrysAlis PRO*; Agilent, 2010 ▶) *T*_min_ = 0.218, *T*_max_ = 0.353
6906 measured reflections
4462 independent reflections
4281 reflections with *I* > 2σ(*I*)
*R*_int_ = 0.039

#### Refinement

*R*[*F* ^2^ > 2σ(*F* ^2^)] = 0.049
*wR*(*F* ^2^) = 0.139
*S* = 1.11
4462 reflections
293 parameters
H-atom parameters constrained
Δρ_max_ = 1.60 e Å^−3^
Δρ_min_ = −1.38 e Å^−3^

### 

Data collection: *CrysAlis PRO* (Agilent, 2010 ▶); cell refinement: *CrysAlis PRO*; data reduction: *CrysAlis PRO*; program(s) used to solve structure: *SHELXS97* (Sheldrick, 2008 ▶); program(s) used to refine structure: *SHELXL97* (Sheldrick, 2008 ▶); molecular graphics: *ORTEP-3* (Farrugia, 1997 ▶), *DIAMOND* (Brandenburg, 2006 ▶) and *Qmol* (Gans & Shalloway, 2001 ▶); software used to prepare material for publication: *publCIF* (Westrip, 2010 ▶).

## Supplementary Material

Crystal structure: contains datablock(s) global, I. DOI: 10.1107/S1600536811037044/hb6406sup1.cif

Structure factors: contains datablock(s) I. DOI: 10.1107/S1600536811037044/hb6406Isup2.hkl

Supplementary material file. DOI: 10.1107/S1600536811037044/hb6406Isup3.cml

Additional supplementary materials:  crystallographic information; 3D view; checkCIF report

## Figures and Tables

**Table 1:** Hydrogen-bond geometry (Å, °)

*D*—H⋯*A*	*D*—H	H⋯*A*	*D*⋯*A*	*D*—H⋯*A*
C7—H7⋯O2^i^	0.95	2.56	3.453 (7)	157
C15—H15⋯Br4^ii^	0.95	2.89	3.796 (5)	160
C19—H19⋯O1^iii^	0.95	2.60	3.462 (6)	152

Symmetry codes: (i) 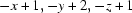; (ii) 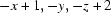; (iii) 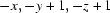.

**Fig. 1.**
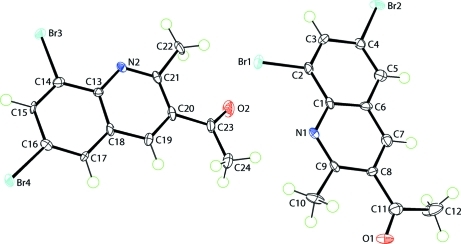
The molecular structures of the two independent molecules comprising the asymmetric unit of (I) showing displacement ellipsoids at the 70% probability level.

**Fig. 2.**
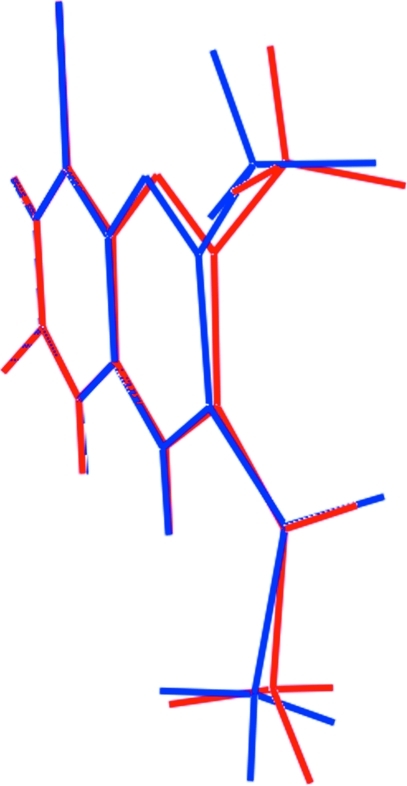
Overlay diagram of the two independent molecules comprising the asymmetric unit of (I). The first independent molecule (with atom S1) is shown in red.

**Fig. 3.**
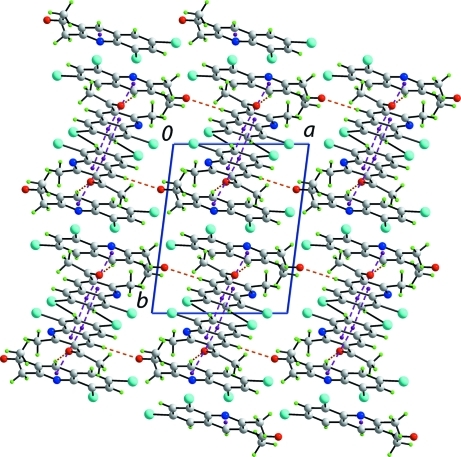
A view in projection down the c axis of the crystal packing in (I) highlighting the stacking of layers along the b axis. The C—H···O and C—H···π interactions are shown as orange and purple dashed lines, respectively.

## supplementary crystallographic information

### Comment

Quinoline derivatives continue to attract wide interest owing to their occurrence in natural products and for their biological activity (Kalluraya & Sreenivasa, 1998; Xiang *et al.*, 2006). In continuation of structural research in this area (Prasath *et al.*, 2011), the title compound, (I), was investigated.

Two independent molecules comprise the crystallographic asymmetric of (I), Fig. 1. The molecules are virtually super-imposable as seen in Fig. 2. The r.m.s. deviations for the bond distances and angles are 0.0088 Å and 0.507 °, respectively (Spek, 2009). The major differences between the molecules are manifested in the values of the C7—C8—C11—C12 and C19—C20—C23—C24 torsion angles of -1.7 (6) and -16.8 (6) °, respectively indicating a twist of the ketone residue out of the plane of the quinolinyl ring in the second independent molecule. In each case, the ketone-O atom is directed towards the ring-methyl group.

In the crystal packing, C—H···O, Table 1, and π–π interactions are noted. The C—H···O and two closest π–π interactions lead to the formation of layers in the *ac* plane. The π–π interactions occur between the (C13–C18) ring and each of the (N1,C1,C6–C9)^i^ [3.584 (3) Å] and (N2,C13,C18–C21)^ii^ [3.615 (3) Å] rings; symmetry operation *i*: 1 - *x*, 1 - *y*, 1 - *z* and *ii*: 1 - *x*, 1 - *y*, 2 - *z*. The resultant layers stack along the *b* axis, Fig. 3.

### Experimental

To a mixture of 2-amino-3,5-dibromobenzaldehyde (0.01 *M*, 2.70 g) and acetylacetone (0.01 *M*, 1.02 ml), 10 ml of 1 N HCl was added. The reaction mixture was stirred at 363 K for 3 h. At the end of this period, the resulting suspension was neutralized with 10 ml of 1 N NaOH. The resultant solid was filtered, dried and purified by column chromatography using a 1:1 mixture of chloroform and hexane. Recrystallization was by slow evaporation of a chloroform solution of (I) which yielded light-brown prisms. Yield: 90%. *M*.pt. 433–435 K.

### Refinement

Carbon-bound H-atoms were placed in calculated positions [C—H 0.95 to 0.98 Å, *U*_iso_(H) = 1.2 to 1.5*U*_eq_(C)] and were included in the refinement in the riding model approximation. The maximum and minimum residual electron density peaks of 1.60 and 1.38 e Å^-3^, respectively, were located 0.93 Å and 0.70 Å from the Br3 and Br2 atoms, respectively.

### Crystal data

**Table:**

C_12_H_9_Br_2_NO	*Z* = 4
*M**_r_* = 343.02	*F*(000) = 664
Triclinic, *P*1	*D*_x_ = 1.987 Mg m^−^^3^
Hall symbol: -P 1	Cu *K*α radiation, λ = 1.54184 Å
*a* = 9.7549 (5) Å	Cell parameters from 4985 reflections
*b* = 11.1719 (6) Å	θ = 3.9–74.1°
*c* = 11.5629 (5) Å	µ = 8.78 mm^−^^1^
α = 99.043 (4)°	*T* = 100 K
β = 93.330 (4)°	Prism, light-brown
γ = 111.733 (5)°	0.25 × 0.20 × 0.15 mm
*V* = 1146.69 (10) Å^3^	

### Data collection

**Table:**

Agilent SuperNova Dual diffractometer with an Atlas detector	4462 independent reflections
Radiation source: SuperNova (Cu) X-ray Source	4281 reflections with *I* > 2σ(*I*)
Mirror	*R*_int_ = 0.039
Detector resolution: 10.4041 pixels mm^-1^	θ_max_ = 74.3°, θ_min_ = 3.9°
ω scans	*h* = −12→12
Absorption correction: multi-scan (*CrysAlis PRO*; Agilent, 2010)	*k* = −13→13
*T*_min_ = 0.218, *T*_max_ = 0.353	*l* = −14→7
6906 measured reflections	

### Refinement

**Table:**

Refinement on *F*^2^	Primary atom site location: structure-invariant direct methods
Least-squares matrix: full	Secondary atom site location: difference Fourier map
*R*[*F*^2^ > 2σ(*F*^2^)] = 0.049	Hydrogen site location: inferred from neighbouring sites
*wR*(*F*^2^) = 0.139	H-atom parameters constrained
*S* = 1.11	*w* = 1/[σ^2^(*F*_o_^2^) + (0.0872*P*)^2^ + 3.5949*P*] where *P* = (*F*_o_^2^ + 2*F*_c_^2^)/3
4462 reflections	(Δ/σ)_max_ = 0.001
293 parameters	Δρ_max_ = 1.60 e Å^−^^3^
0 restraints	Δρ_min_ = −1.38 e Å^−^^3^

### Special details

**Table:**

Geometry. All e.s.d.'s (except the e.s.d. in the dihedral angle between two l.s. planes) are estimated using the full covariance matrix. The cell e.s.d.'s are taken into account individually in the estimation of e.s.d.'s in distances, angles and torsion angles; correlations between e.s.d.'s in cell parameters are only used when they are defined by crystal symmetry. An approximate (isotropic) treatment of cell e.s.d.'s is used for estimating e.s.d.'s involving l.s. planes.
Refinement. Refinement of *F*^2^ against ALL reflections. The weighted *R*-factor *wR* and goodness of fit *S* are based on *F*^2^, conventional *R*-factors *R* are based on *F*, with *F* set to zero for negative *F*^2^. The threshold expression of *F*^2^ > σ(*F*^2^) is used only for calculating *R*-factors(gt) *etc*. and is not relevant to the choice of reflections for refinement. *R*-factors based on *F*^2^ are statistically about twice as large as those based on *F*, and *R*- factors based on ALL data will be even larger.

### Fractional atomic coordinates and isotropic or equivalent isotropic displacement parameters (Å^2^)

**Table:**

	*x*	*y*	*z*	*U*_iso_*/*U*_eq_	
Br1	0.64524 (5)	0.65200 (5)	0.49508 (4)	0.01426 (16)	
Br2	0.92320 (5)	1.16226 (5)	0.40306 (4)	0.01406 (16)	
Br3	0.85223 (5)	0.34850 (5)	1.00127 (4)	0.01197 (15)	
Br4	0.24944 (5)	0.00166 (4)	0.93076 (4)	0.01288 (15)	
O1	−0.0502 (4)	0.7000 (4)	0.2695 (3)	0.0221 (8)	
O2	0.5755 (4)	0.8053 (4)	0.7753 (3)	0.0192 (8)	
N1	0.3618 (4)	0.6815 (4)	0.3952 (3)	0.0095 (7)	
N2	0.7242 (4)	0.5237 (4)	0.8901 (3)	0.0081 (7)	
C1	0.4870 (5)	0.7915 (4)	0.3957 (4)	0.0080 (8)	
C2	0.6289 (5)	0.7963 (4)	0.4388 (4)	0.0096 (8)	
C3	0.7562 (5)	0.9045 (5)	0.4393 (4)	0.0112 (9)	
H3	0.8503	0.9061	0.4678	0.013*	
C4	0.7460 (5)	1.0134 (4)	0.3971 (4)	0.0092 (9)	
C5	0.6127 (5)	1.0128 (5)	0.3557 (4)	0.0134 (9)	
H5	0.6077	1.0865	0.3271	0.016*	
C6	0.4816 (5)	0.9023 (5)	0.3553 (4)	0.0107 (9)	
C7	0.3414 (5)	0.8974 (5)	0.3137 (4)	0.0117 (9)	
H7	0.3340	0.9701	0.2849	0.014*	
C8	0.2144 (5)	0.7890 (5)	0.3138 (4)	0.0103 (9)	
C9	0.2302 (5)	0.6785 (4)	0.3544 (4)	0.0084 (8)	
C10	0.1013 (5)	0.5530 (5)	0.3547 (4)	0.0165 (10)	
H10A	0.1373	0.4915	0.3852	0.025*	
H10B	0.0511	0.5143	0.2740	0.025*	
H10C	0.0311	0.5710	0.4051	0.025*	
C11	0.0666 (5)	0.7922 (5)	0.2724 (4)	0.0141 (10)	
C12	0.0674 (6)	0.9161 (5)	0.2366 (6)	0.0250 (12)	
H12A	−0.0349	0.9111	0.2240	0.037*	
H12B	0.1125	0.9265	0.1634	0.037*	
H12C	0.1252	0.9916	0.2992	0.037*	
C13	0.6151 (5)	0.4096 (4)	0.9012 (4)	0.0071 (8)	
C14	0.6509 (5)	0.3130 (5)	0.9490 (4)	0.0095 (8)	
C15	0.5430 (5)	0.1943 (4)	0.9581 (4)	0.0099 (8)	
H15	0.5698	0.1309	0.9890	0.012*	
C16	0.3936 (5)	0.1679 (5)	0.9212 (4)	0.0104 (9)	
C17	0.3516 (5)	0.2581 (4)	0.8784 (4)	0.0084 (8)	
H17	0.2495	0.2398	0.8563	0.010*	
C18	0.4625 (5)	0.3792 (4)	0.8676 (4)	0.0093 (8)	
C19	0.4264 (5)	0.4746 (4)	0.8214 (4)	0.0089 (8)	
H19	0.3252	0.4584	0.7980	0.011*	
C20	0.5357 (5)	0.5907 (4)	0.8098 (4)	0.0097 (8)	
C21	0.6871 (5)	0.6116 (4)	0.8459 (4)	0.0093 (8)	
C22	0.8165 (5)	0.7332 (5)	0.8342 (4)	0.0146 (9)	
H22A	0.9094	0.7198	0.8477	0.022*	
H22B	0.8186	0.8079	0.8925	0.022*	
H22C	0.8057	0.7512	0.7546	0.022*	
C23	0.4916 (6)	0.6915 (5)	0.7634 (4)	0.0134 (9)	
C24	0.3351 (6)	0.6467 (5)	0.7000 (4)	0.0173 (10)	
H24A	0.3369	0.6938	0.6351	0.026*	
H24B	0.2702	0.6652	0.7557	0.026*	
H24C	0.2972	0.5521	0.6685	0.026*	

### Atomic displacement parameters (Å^2^)

**Table:**

	*U*^11^	*U*^22^	*U*^33^	*U*^12^	*U*^13^	*U*^23^
Br1	0.0141 (3)	0.0157 (3)	0.0155 (3)	0.0064 (2)	−0.00005 (19)	0.0089 (2)
Br2	0.0070 (3)	0.0118 (3)	0.0201 (3)	−0.0001 (2)	0.00311 (18)	0.0024 (2)
Br3	0.0070 (3)	0.0125 (3)	0.0165 (3)	0.0031 (2)	−0.00128 (18)	0.00586 (19)
Br4	0.0103 (3)	0.0077 (3)	0.0200 (3)	0.00088 (19)	0.00047 (18)	0.00814 (19)
O1	0.0065 (16)	0.029 (2)	0.029 (2)	0.0018 (15)	0.0026 (14)	0.0116 (16)
O2	0.0233 (19)	0.0126 (17)	0.0236 (18)	0.0072 (15)	0.0012 (15)	0.0084 (14)
N1	0.0104 (18)	0.0121 (19)	0.0070 (16)	0.0043 (15)	0.0040 (14)	0.0041 (14)
N2	0.0091 (17)	0.0099 (18)	0.0050 (16)	0.0028 (15)	0.0022 (13)	0.0020 (14)
C1	0.012 (2)	0.009 (2)	0.0046 (18)	0.0048 (18)	0.0032 (15)	0.0024 (15)
C2	0.012 (2)	0.012 (2)	0.0048 (18)	0.0042 (18)	0.0013 (15)	0.0031 (16)
C3	0.009 (2)	0.015 (2)	0.012 (2)	0.0071 (18)	0.0010 (16)	0.0048 (17)
C4	0.010 (2)	0.007 (2)	0.011 (2)	0.0027 (17)	0.0027 (16)	0.0020 (16)
C5	0.012 (2)	0.014 (2)	0.015 (2)	0.0051 (19)	0.0035 (17)	0.0044 (18)
C6	0.007 (2)	0.015 (2)	0.0092 (19)	0.0044 (18)	0.0032 (16)	0.0018 (17)
C7	0.015 (2)	0.013 (2)	0.011 (2)	0.0076 (19)	0.0046 (17)	0.0044 (17)
C8	0.007 (2)	0.017 (2)	0.0076 (19)	0.0062 (18)	0.0015 (15)	0.0032 (17)
C9	0.010 (2)	0.010 (2)	0.0053 (18)	0.0023 (18)	0.0031 (15)	0.0031 (16)
C10	0.008 (2)	0.015 (2)	0.018 (2)	−0.0032 (19)	0.0038 (18)	−0.0005 (19)
C11	0.012 (2)	0.019 (2)	0.011 (2)	0.006 (2)	0.0016 (17)	0.0012 (18)
C12	0.013 (2)	0.017 (3)	0.050 (4)	0.010 (2)	0.003 (2)	0.008 (2)
C13	0.0066 (19)	0.008 (2)	0.0073 (18)	0.0032 (17)	0.0016 (15)	0.0015 (15)
C14	0.008 (2)	0.015 (2)	0.0061 (19)	0.0050 (18)	0.0001 (15)	0.0017 (16)
C15	0.012 (2)	0.0070 (19)	0.014 (2)	0.0060 (18)	0.0008 (17)	0.0062 (16)
C16	0.009 (2)	0.009 (2)	0.012 (2)	0.0013 (18)	0.0022 (16)	0.0028 (17)
C17	0.007 (2)	0.009 (2)	0.011 (2)	0.0048 (17)	0.0006 (15)	0.0047 (16)
C18	0.014 (2)	0.012 (2)	0.0048 (18)	0.0062 (18)	0.0027 (16)	0.0047 (16)
C19	0.013 (2)	0.009 (2)	0.0063 (19)	0.0060 (18)	0.0003 (16)	0.0017 (16)
C20	0.018 (2)	0.008 (2)	0.0054 (18)	0.0063 (18)	0.0031 (16)	0.0024 (16)
C21	0.014 (2)	0.010 (2)	0.0042 (18)	0.0040 (18)	0.0036 (16)	0.0026 (16)
C22	0.014 (2)	0.012 (2)	0.016 (2)	0.0012 (19)	0.0046 (18)	0.0093 (18)
C23	0.019 (2)	0.016 (2)	0.011 (2)	0.010 (2)	0.0049 (18)	0.0086 (18)
C24	0.018 (2)	0.016 (2)	0.021 (2)	0.008 (2)	0.0006 (19)	0.0103 (19)

### Geometric parameters (Å, °)

**Table:**

Br1—C2	1.886 (5)	C10—H10C	0.9800
Br2—C4	1.892 (5)	C11—C12	1.503 (7)
Br3—C14	1.895 (4)	C12—H12A	0.9800
Br4—C16	1.894 (5)	C12—H12B	0.9800
O1—C11	1.215 (6)	C12—H12C	0.9800
O2—C23	1.211 (6)	C13—C18	1.414 (6)
N1—C9	1.328 (6)	C13—C14	1.426 (6)
N1—C1	1.374 (6)	C14—C15	1.379 (6)
N2—C21	1.324 (6)	C15—C16	1.401 (6)
N2—C13	1.355 (6)	C15—H15	0.9500
C1—C6	1.407 (6)	C16—C17	1.366 (6)
C1—C2	1.422 (6)	C17—C18	1.415 (6)
C2—C3	1.374 (7)	C17—H17	0.9500
C3—C4	1.413 (6)	C18—C19	1.407 (6)
C3—H3	0.9500	C19—C20	1.373 (6)
C4—C5	1.357 (7)	C19—H19	0.9500
C5—C6	1.410 (7)	C20—C21	1.433 (7)
C5—H5	0.9500	C20—C23	1.505 (6)
C6—C7	1.402 (7)	C21—C22	1.505 (6)
C7—C8	1.375 (7)	C22—H22A	0.9800
C7—H7	0.9500	C22—H22B	0.9800
C8—C9	1.443 (6)	C22—H22C	0.9800
C8—C11	1.508 (6)	C23—C24	1.519 (7)
C9—C10	1.499 (6)	C24—H24A	0.9800
C10—H10A	0.9800	C24—H24B	0.9800
C10—H10B	0.9800	C24—H24C	0.9800
			
C9—N1—C1	119.1 (4)	H12B—C12—H12C	109.5
C21—N2—C13	118.9 (4)	N2—C13—C18	123.0 (4)
N1—C1—C6	122.5 (4)	N2—C13—C14	120.4 (4)
N1—C1—C2	119.9 (4)	C18—C13—C14	116.5 (4)
C6—C1—C2	117.6 (4)	C15—C14—C13	121.8 (4)
C3—C2—C1	121.2 (4)	C15—C14—Br3	119.0 (3)
C3—C2—Br1	118.7 (3)	C13—C14—Br3	119.2 (3)
C1—C2—Br1	120.1 (3)	C14—C15—C16	119.4 (4)
C2—C3—C4	119.5 (4)	C14—C15—H15	120.3
C2—C3—H3	120.3	C16—C15—H15	120.3
C4—C3—H3	120.3	C17—C16—C15	121.6 (4)
C5—C4—C3	121.1 (4)	C17—C16—Br4	120.3 (4)
C5—C4—Br2	120.6 (4)	C15—C16—Br4	118.1 (3)
C3—C4—Br2	118.2 (3)	C16—C17—C18	119.0 (4)
C4—C5—C6	119.7 (4)	C16—C17—H17	120.5
C4—C5—H5	120.2	C18—C17—H17	120.5
C6—C5—H5	120.2	C19—C18—C17	121.6 (4)
C7—C6—C1	117.3 (4)	C19—C18—C13	116.8 (4)
C7—C6—C5	121.7 (4)	C17—C18—C13	121.6 (4)
C1—C6—C5	121.0 (4)	C20—C19—C18	120.8 (4)
C8—C7—C6	121.1 (4)	C20—C19—H19	119.6
C8—C7—H7	119.4	C18—C19—H19	119.6
C6—C7—H7	119.4	C19—C20—C21	118.1 (4)
C7—C8—C9	118.0 (4)	C19—C20—C23	118.9 (4)
C7—C8—C11	118.4 (4)	C21—C20—C23	122.9 (4)
C9—C8—C11	123.6 (4)	N2—C21—C20	122.4 (4)
N1—C9—C8	121.9 (4)	N2—C21—C22	114.8 (4)
N1—C9—C10	114.9 (4)	C20—C21—C22	122.8 (4)
C8—C9—C10	123.2 (4)	C21—C22—H22A	109.5
C9—C10—H10A	109.5	C21—C22—H22B	109.5
C9—C10—H10B	109.5	H22A—C22—H22B	109.5
H10A—C10—H10B	109.5	C21—C22—H22C	109.5
C9—C10—H10C	109.5	H22A—C22—H22C	109.5
H10A—C10—H10C	109.5	H22B—C22—H22C	109.5
H10B—C10—H10C	109.5	O2—C23—C20	122.5 (4)
O1—C11—C12	120.3 (5)	O2—C23—C24	119.7 (4)
O1—C11—C8	122.1 (5)	C20—C23—C24	117.8 (4)
C12—C11—C8	117.5 (4)	C23—C24—H24A	109.5
C11—C12—H12A	109.5	C23—C24—H24B	109.5
C11—C12—H12B	109.5	H24A—C24—H24B	109.5
H12A—C12—H12B	109.5	C23—C24—H24C	109.5
C11—C12—H12C	109.5	H24A—C24—H24C	109.5
H12A—C12—H12C	109.5	H24B—C24—H24C	109.5
			
C9—N1—C1—C6	0.4 (6)	C21—N2—C13—C18	0.2 (6)
C9—N1—C1—C2	−179.8 (4)	C21—N2—C13—C14	−179.6 (4)
N1—C1—C2—C3	179.3 (4)	N2—C13—C14—C15	−177.9 (4)
C6—C1—C2—C3	−0.9 (6)	C18—C13—C14—C15	2.3 (6)
N1—C1—C2—Br1	0.0 (5)	N2—C13—C14—Br3	1.9 (5)
C6—C1—C2—Br1	179.8 (3)	C18—C13—C14—Br3	−177.9 (3)
C1—C2—C3—C4	0.5 (6)	C13—C14—C15—C16	−1.1 (7)
Br1—C2—C3—C4	179.8 (3)	Br3—C14—C15—C16	179.1 (3)
C2—C3—C4—C5	−0.2 (7)	C14—C15—C16—C17	−1.2 (7)
C2—C3—C4—Br2	178.4 (3)	C14—C15—C16—Br4	178.2 (3)
C3—C4—C5—C6	0.4 (7)	C15—C16—C17—C18	2.1 (7)
Br2—C4—C5—C6	−178.1 (3)	Br4—C16—C17—C18	−177.3 (3)
N1—C1—C6—C7	0.2 (6)	C16—C17—C18—C19	178.4 (4)
C2—C1—C6—C7	−179.6 (4)	C16—C17—C18—C13	−0.8 (6)
N1—C1—C6—C5	−179.2 (4)	N2—C13—C18—C19	−0.4 (6)
C2—C1—C6—C5	1.1 (6)	C14—C13—C18—C19	179.4 (4)
C4—C5—C6—C7	179.8 (4)	N2—C13—C18—C17	178.8 (4)
C4—C5—C6—C1	−0.9 (7)	C14—C13—C18—C17	−1.3 (6)
C1—C6—C7—C8	0.7 (7)	C17—C18—C19—C20	−179.0 (4)
C5—C6—C7—C8	−180.0 (4)	C13—C18—C19—C20	0.3 (6)
C6—C7—C8—C9	−2.0 (6)	C18—C19—C20—C21	0.1 (6)
C6—C7—C8—C11	177.6 (4)	C18—C19—C20—C23	−178.2 (4)
C1—N1—C9—C8	−1.9 (6)	C13—N2—C21—C20	0.2 (6)
C1—N1—C9—C10	178.5 (4)	C13—N2—C21—C22	−178.4 (4)
C7—C8—C9—N1	2.7 (6)	C19—C20—C21—N2	−0.4 (6)
C11—C8—C9—N1	−176.9 (4)	C23—C20—C21—N2	177.9 (4)
C7—C8—C9—C10	−177.7 (4)	C19—C20—C21—C22	178.2 (4)
C11—C8—C9—C10	2.7 (7)	C23—C20—C21—C22	−3.6 (6)
C7—C8—C11—O1	179.7 (4)	C19—C20—C23—O2	162.9 (5)
C9—C8—C11—O1	−0.7 (7)	C21—C20—C23—O2	−15.4 (7)
C7—C8—C11—C12	−1.7 (6)	C19—C20—C23—C24	−16.8 (6)
C9—C8—C11—C12	177.9 (4)	C21—C20—C23—C24	165.0 (4)

### Hydrogen-bond geometry (Å, °)

**Table:**

*D*—H···*A*	*D*—H	H···*A*	*D*···*A*	*D*—H···*A*
C7—H7···O2^i^	0.95	2.56	3.453 (7)	157
C15—H15···Br4^ii^	0.95	2.89	3.796 (5)	160
C19—H19···O1^iii^	0.95	2.60	3.462 (6)	152

Symmetry codes: (i) −*x*+1, −*y*+2, −*z*+1; (ii) −*x*+1, −*y*, −*z*+2; (iii) −*x*, −*y*+1, −*z*+1.
